# Health outcomes, education, healthcare delivery and quality – 3048. From uterus to university: Recruitment and retention of a primary prevention birth cohort

**DOI:** 10.1186/1939-4551-6-S1-P218

**Published:** 2013-04-23

**Authors:** Brenda Gerwing, Rishma Chooniedass, Saiful Huq, Hao Huang, Anita Kozyrskyj, Edmond Chan, Clare Ramsey, Moira Chan-Yeung, Allan Becker

**Affiliations:** 1 Pediatrics and Child Health, Section of Allergy, University of Manitoba, Canada; 2 University of Manitoba, Winnipeg, MB, Canada; 3 Pediatrics, University of Alberta, Edmonton, AB, Canada; 4 Pediatrics, University of British Columbia, Vancouver, BC, Canada; 5 Internal Medicine, University of Manitoba, Winnipeg, MB, Canada; 6 Internal Medicine, University of British Columbia, Vancouver, BC, Canada

## Background

It is important to identify predictors of retention in primary prevention studies as recruitment and retention are critical factors for a successful intervention study.

## Methods

In 1994, the Canadian Asthma Primary Prevention Study (CAPPS) was established. This high-risk birth cohort has 2 sites, Winnipeg and Vancouver, Canada. Expectant mothers were recruited during the third trimester. Enrollment criteria were a first degree relative with asthma or two first degree relatives with other allergic diseases. Participants were prenatally randomized into control and intervention groups. Intervention measures were introduced before birth and during baby’s first year of life. Follow-up assessments by a Pediatric Allergist included skin prick testing (SPT) to common food and inhalants and pulmonary function testing.

## Results

545 participants initially recruited. 266 randomized into control and 279 intervention. From recruitment to first year, 9.5% families (52) discontinued. At age 1, 493 infants were assessed; 52.3% males and 47.7% females, 49.1% control and 50.9% intervention. 76.8% high SES, 22.52% low SES. 9.7% maternal age ≤25 and 90.3% maternal age >25. 17.6 % were diagnosed with asthma at 1 year. 22.1% with +SPT to food. 44.2% were 1^st^born. Children were assessed at 2 (n=472, 95.7%) and 7 years (n=380, 77.1%). At 15 years, 326 (66.1%) participants returned; 55.8% males and 44.2% females (p=0.02), 44.5% control and 54.6% intervention (p=0.054). Maternal age >25 (OR=1.73, 95% CI 0.95-3.16, p=0.05), asthma diagnosis (OR=1.53, 95% CI 0.91-2.57, p=0.066), high SES (OR=1.37, 95% CI 0.88-2.11, p=0.1) and +SPT (OR=1.23, 95% CI 0.78-1.95, p=0.22) were all associated with higher rates of return. While 138 participants returned with no sibling(s) at enrollment (OR=0.81, 95% CI 0.56-1.18, p=0.16).

## Conclusions

Participants with sibling(s) at birth had no significant difference in retention. Maternal age was the most likely predictor of participant drop out. Female participants, low SES, negative skin prick test to food and no asthma diagnosis at age 1 showed a trend towards drop out. When establishing future asthma and allergy cohorts, specific retention strategies should be considered for groups identified at risk for drop out, especially for younger mothers and female participants.

